# Comparative analysis of methods for diagnosing successful aging and quality of life in the elderly (on example of a Russian sample)

**DOI:** 10.1192/j.eurpsy.2026.12218

**Published:** 2026-07-31

**Authors:** S. D. Tomilovskiy, A. M. Konovalova, V. D. Vinogradova

**Affiliations:** 1 Faculty of Psychology, Lomonosov Moscow State University; 2 Department of Pedagogy and Medical Psychology, Sechenov First Moscow State Medical University; 3 Department of Pedagogy and Medical Psychology, I.M.Sechenov First Moscow State Medical University of the Ministry of Health of the Russian Federation (Sechenovskiy University), Moscow, Russian Federation

## Abstract

**Introduction:**

Since the second half of the 20th century, the problems of active/effective/successful aging, quality of life have been actively studied and methods for their diagnostics have been developed

**Objectives:**

To conduct a comparative analysis of methods for diagnosing successful aging and quality of life in the elderly using a Russian sample.

**Methods:**

A pilot study was conducted. Four methods were used in this study: APQ (Attitudes to Ageing Questionnaire) (M. Barker, 2007, adoption M.D. Petrash М, O. Yu. Strizhitskaya, 2024); LSITA-SF (Life Satisfaction Index for the Third Age – Short Form) (A. Barrett, 2009, adoption A.A. Zolotaryova et al., 2022); WHOQOL-100 (World Health Organization Quality of Life Assessment) (WHO, 1998); SF-36 (Short Form-36 Health Survey) (Ware, Sherbourne, 1992, adoption A.B. Leonova, 2014).

The study sample consists of 60 elderly people from 52 to 70 years of age (M = 59,5). Among them women accounted for 58,3% (n=35), men 41,7 % (n=25).

**Results:**

Here is a short description of each of the methods with their comparison on the main characteristics. 1. APQ: This methodology is based on the assumption that perception of ageing is a significant predictor of psychological well-being in older adults. It identifies how an individual interprets age-related changes – as a loss or as a source of new opportunities. The instrument includes several scales reflecting different aspects of attitudes towards ageing. This self-report questionnaire consists of 31 items (~11 min). 2. LSITA-SF: This methodology is designed to assess the subjective feeling of life satisfaction among older adults, particularly relevant in studies of quality of life, psychoemotional state, and successful ageing. The short form of the index contains 12 statements (~2 min). 3. WHOQOL-100: In this questionnaire, quality of life is defined as an individual’s subjective perception of their position in life within the context of their culture, value system, goals, and expectations. A comprehensive assessment of quality of life is conducted across six domains. It includes 100 items across 6 domains and 24 facets (~18 min). 4. SF-36: This tool provides a concise yet informative overview of physical and mental health, including health-related quality of life. It comprises 36 items grouped into 8 scales (~7 min).

**Conclusions:**

The methods used have the following advantages and limitations.

APQ covers both positive and negative attitudes but does not measure physical or social functioning. LSITA-SF allows for a quick and easy assessment of subjective well-being, but provides only a general assessment without a breakdown into scales. WHOQOL-100. Pros: multi-faceted, high precision. Cons: difficult for the elderly to perform. SF-36 convenient, short, valid for mass research, but does not consider the spiritual-existential sphere. The pilot study showed a high degree of agreement between these methods.

**Disclosure of Interest:**

None Declared

